# A 3D Printing-Based Transcatheter Pulmonary Valve Replacement Simulator: Development and Validation

**DOI:** 10.3390/bioengineering12040344

**Published:** 2025-03-26

**Authors:** Yuanzhang Liu, Yu Mao, Yiwei Wang, Ping Jin, Mengen Zhai, Yang Liu, Jian Yang

**Affiliations:** Department of Cardiovascular Surgery, Xijing Hospital, Air Force Medical University, Xi’an 710032, China; liuyz1222@126.com (Y.L.);

**Keywords:** cardiovascular 3D printing, transcatheter pulmonary valve replacement, simulation, training

## Abstract

Background: Severe pulmonary regurgitation (PR) often occurs after treatment of tetralogy of Fallot with a valve ring patch, leading to enlargement and diverse morphological characteristics of the native right ventricular outflow tract (nRVOT), which increases the difficulty of transcatheter pulmonary valve replacement (TPVR). The purpose of this study was to use the TPVR simulator to help doctors improve their surgical skills by simulating the surgical process in vitro. Methods: The TPVR simulator was developed using three-dimensional (3D) printing technology under computer-aided design. In this study, the TPVR simulator was used for preoperative simulation training and teaching. First, 10 specialists were equally divided into a 3D-printed group and a non-3D-printed group, each performing one TPVR; then, another six specialists and six young surgeons were selected to complete three TPVR simulations. Results: For the 3D-printed simulation group, the over-flap time (5.22 min (range: 4.85–5.87 min) vs. 6.72 min (range: 6.12–7.70 min), *p* = 0.016), fluoroscopy time (15.00 min (range: 13.50–16.50 min) vs. 19.00 min (range: 17.50–21.50 min), *p* = 0.012), and total operative time for the five surgeons (57.00 min (range: 54.00–62.50 min) vs. 67.00 min (range: 62.00–69.50 min), *p* = 0.036) were shorter. In addition, the results showed significant reductions in the median over-flap time and total time required in both the expert panel and young surgeon groups (all *p* < 0.05). Conclusions: The reliability and validity of the TPVR simulator was initially demonstrated and has the potential to be a teaching and training tool for surgeons.

## 1. Introduction

Severe pulmonary regurgitation (PR) represents the most prevalent lesion in the aftermath of tetralogy of Fallot treatment, manifesting in both pediatric and adult patients. The clinical presentation is characterized by right ventricular dilatation and dysfunction, the occurrence of arrhythmias, and limitations in exercise capacity [1,2]. Transcatheter pulmonary valve replacement (TPVR) has been demonstrated to be a promising treatment option for PR. It has been shown to reduce the need for additional surgical intervention in PR patients [3,4,5,6]. It is unfortunate that approximately 80% of patients undergoing surgical RVOT reconstruction receive either retained valve repair or transannular patching [7]. Not only does this treatment lead to severe PR later in the patient’s life, but it also results in a variety of morphological features of the nRVOT that increase the surgical difficulty of TPVR [8].

As medical visualization continues to evolve, there is an increasing demand for accuracy in digital modeling. This demand is particularly pronounced in the field of cardiovascular disease, where precision is of the essence [9]. In recent years, three-dimensional (3D) printing has emerged as a technology that is both user-friendly and versatile. This is a technology application that prints a corresponding model that exactly matches the reconstructed model, layer by layer, based on 3D reconstruction data formed by medical imaging scans, providing surgeons with a personalized model that helps them understand anatomy at a glance [10]. As 3D printing technology continues to improve, there are anecdotal reports that it can help in the implementation of TPVR [11,12]. Furthermore, patient-specific 3D-printed models can be used to simulate preoperative conditions to assess the risk of complications such as coronary compression and perivalvular leakage, and to develop a personalized preoperative plan that can help improve the safety and effectiveness of surgery [13,14].

PR is a major disease that affects the quality of life of patients. Improvements in its treatment are crucial to improving patient outcomes. The objective of this research is to utilize a TPVR simulator to help doctors improve their surgical skills by simulating the in vitro surgical process, while filling the current research gap in the application of 3D printing technology in TPVR training. By converting patient CT data into an actionable 3D model, a realistic simulation training environment is provided for young cardiovascular specialists, thereby improving surgical outcomes and the overall prognosis of patients. The research methods mainly include the production of 3D-printed models and their application in surgical training, with a focus on evaluating the effectiveness of this technology in surgical training and comparing it with traditional training methods. Through rigorous research design and data analysis, this study hopes to provide an empirical basis for the application of 3D printing technology in TPVR training.

## 2. Materials and Methods

### 2.1. 3D Printing the RVOT Model Process and Building the TPVR Simulator

The Digital Imaging and Communications in Medicine (DICOM) format of the computed tomography (CT) data of patients with PR was imported into Materialise Mimics Version 21.0 software (Leuven, Belgium). Utilizing the threshold segmentation feature, a three-dimensional reconstruction of the RVOT was generated at the conclusion of the contraction phase. Subsequently, the resulting 3D model was further processed in Materialise 3-Matic Version 13.0 software (Leuven, Belgium), where it underwent extraction, cropping, smoothing, and repairs to accurately restore the anatomical structures in a one-to-one representation. Further, the standard triangular language files of the 3D reconstructions were processed. The model was exported to a Stratasys Polyjet 850 multi-material full-color 3D printer. Specific right ventricle–pulmonary artery (RV-PA) models operate by printing right ventricular and pulmonary artery tissue using rubber-like flexible resin material (tensile strength: 1.95 MPa, elongation at break: 190%, compression strength: 0.300 MPa, shore hardness: 24 Shore A, Stratasys, Israel) and printing calcified tissue using rigid resin material (tensile strength: 31.7 MPa, elongation at break: 25%, flexural strength: 41.4 MPa, yield compression stress: 37.6 MPa, shore hardness: 82 Shore D, Stratasys, Israel).

**Figure 1:**
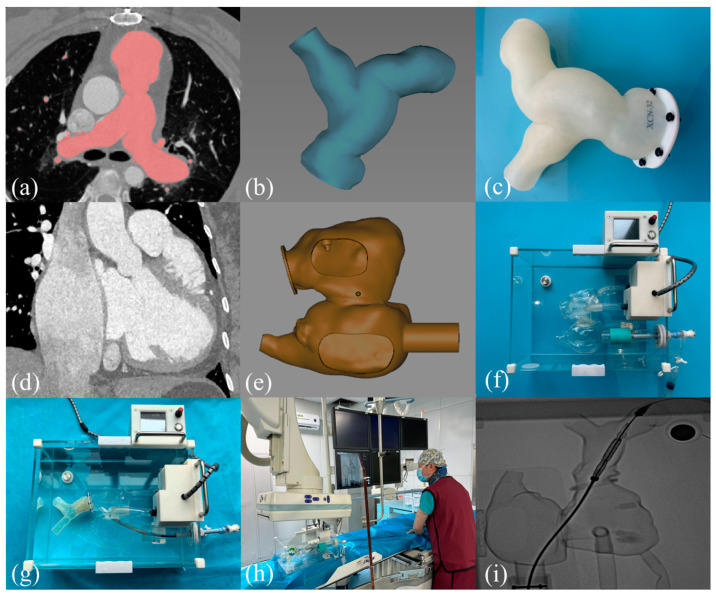
Construction of the TPVR simulator and simulation training on the bench test. (**a**–**c**) The main process of 3D-printed RV-PA models; (**d**–**f**) The main process of the pulsatile simulator; (**g**–**i**) Trainees simulated TPVR using the pulsatile simulator. TPVR, transcatheter pulmonary valve replacement; 3D, three-dimensional; RV-PA, right ventricle–pulmonary artery.

To construct the TPVR simulator, the patient’s CT data were first divided into three parts—inferior vena cava, right atrium, and right ventricle—to measure the internal diameter and the corresponding curvature. Utilizing the acquired measurements, specialized engineers proceeded to fabricate the respective components of the 3D reconstructed model and meticulously designed the grommets to ensure a secure fit for each segment. The simulator primarily comprised two components: (1) the operational part, which encompassed the transfemoral vein along with the 3D-printed RV-PA model, and (2) the driving part, which featured the circulatory pump, the fully integrated connection loop, and the control mechanism. By creating a pulsatile simulation that mimics pressure–pulsatile blood flow, the 3D-printed RV-PA model, together with the surrounding tissues, can simulate movement under realistic pathophysiological conditions (Figure 1).

### 2.2. Guidance for 3D Printing

For the professional development of young cardiovascular specialists, simulation training is essential. With the rapid evolution of computer graphics, bioengineering, and digital modeling technologies, 3D printing-based TPVR training systems will lead to more simulation-based training options. Trainees can practice several major pre-dural steps (such as crossing the valve, exchanging threads, positioning the stent) by using specific 3D-printed models to improve their manipulation skills and proficiency for an improved learning curve. It can also help doctors select valves by simulating placement to assess the risk of coronary artery compression, valve migration, and vascular complications.

### 2.3. The 3D-Printed Group Versus the Non-3D-Printed Group 

**Figure 2:**
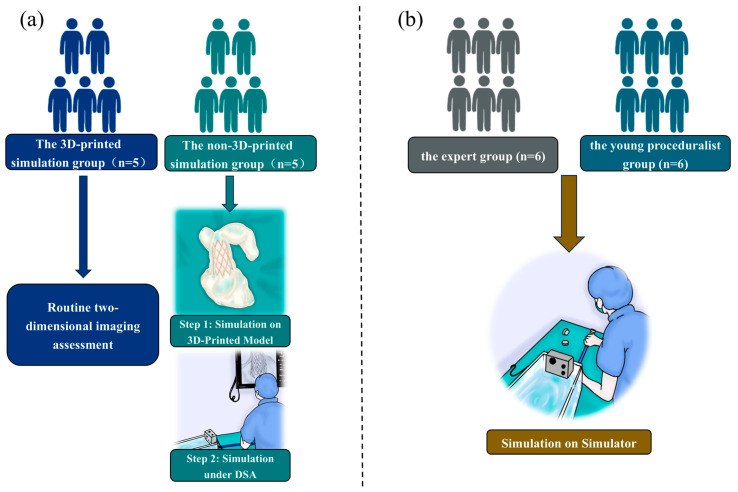
Schematic of the flow of this study. (**a**) The five proceduralists in the group utilizing 3D-printed simulations sequentially performed two simulation steps prior to the TPVR. In contrast, the five proceduralists in the non-3D-printed simulation group carried out a standard two-dimensional imaging evaluation before the TPVR procedure. (**b**) Both the expert group (*n* = 6) and the young proceduralist group (*n* = 6) successfully conducted three rounds of simulations using the pulsatile simulator.

The flow diagram of the entire study is shown in Figure 2. A total of ten patients diagnosed with severe PR were systematically categorized into two groups: a 3D-printed simulation group and a non-3D-printed simulation group. Each group consisted of five patients. In parallel, ten specialists were selected and evenly divided into two cohorts. Within the 3D-printed simulation group, five proceduralists practiced the procedural steps through a bench test prior to TPVR. Conversely, the proceduralists in the non-3D-printed simulation group proceeded with TPVR following standard preoperative assessments. In this study, the Venus P-valve™ (Venus Medtech, Hangzhou, China) was used for both preoperative simulation and intraoperative use in TPVR. Each expert completed one operation, and the crossing-valve time, fluoroscopy time, total time, and the occurrence of the major postoperative complications were recorded and statistically analyzed. This study was reviewed and approved by the Ethics Committee of Xijing Hospital (KY20192138-C1) and conducted in accordance with the principles of the Declaration of Helsinki. The ethical registration was entered in the ClinicalTrials.gov Protocol Registration System (NCT02917980; 27 September 2016). All activities mentioned above were performed within the context of structural heart disease, specifically concentrating on valvular heart disease.

### 2.4. Experts Versus Young Surgeons in the Simulator 

In addition, a total of six experts and six junior proceduralists were chosen and allocated into two distinct groups: one comprising the experts and the other consisting of young proceduralists. Each group contained six individuals. Every participant, whether an expert or a junior proceduralist, underwent three simulation sessions, during which the time taken to cross the valve and the overall duration of the simulations were meticulously documented. The outcomes from the first, second, and third simulation sessions were subsequently compared in pairs and subjected to statistical analysis.

### 2.5. Statistical Analysis

All statistical evaluations were conducted utilizing the Statistical Package for the Social Sciences (SPSS, Chicago, IL, USA), version 26.0. Continuous variables were expressed as the mean, standard deviation, or median within the interquartile range. Normally distributed data were compared using t-tests and non-normally distributed data were compared using non-parametric tests. A two-sided *p*-value of less than 0.05 was deemed statistically significant.

## 3. Results

### 3.1. The 3D-Printed Group Versus the Non-3D-Printed Group 

**Figure 3:**
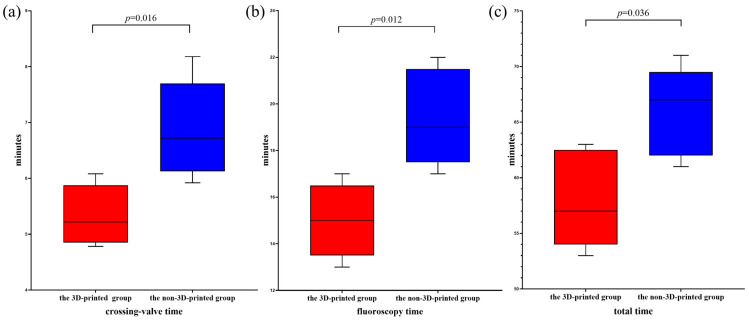
Outcomes of TPVR. (**a**) A comparative analysis of the crossing-valve duration was conducted between the group utilizing 3D-printed simulations and the group employing non-3D-printed simulations, revealing a statistically significant difference (*p* = 0.016). (**b**) An additional comparison of the crossing-valve duration between the 3D-printed simulation cohort and the non-3D-printed simulation cohort indicated a significant difference in fluoroscopy time as well (*p* = 0.012). (**c**) Furthermore, a comparison of the overall procedural time between the 3D-printed simulation group and the non-3D-printed simulation group demonstrated a highly significant difference (*p* = 0.036). The *p*-value is from a non-parametric test.

A cohort of ten patients diagnosed with pulmonary valve regurgitation was selected from Xijing Hospital for this study. This group comprised four males and six females, with an average age of 30.50 years (range: 21.25–43.50 years). The baseline characteristics of these patients are detailed in Table 1. Table 2 presents the baseline characteristics of the proceduralists involved in this study. Within the 3D-printed simulation cohort, the average age was recorded at 42.00 years (range: 38.50–44.50 years), while the mean years of professional experience were 9.00 years (range: 7.50–10.50 years) for interventions. Conversely, in the non-3D-printed simulation cohort, the average age was 42.00 years (range: 38.50–44.00 years), with an intervention experience of 8.00 years (range: 7.50–10.50 years). All ten specialists successfully performed one procedure each, with intraoperative data presented in Table 3. In the non-3D-printed simulation cohort, the mean over-the-valve time for the five surgeons was 6.72 min (range: 6.12–7.70 min), the fluoroscopy time averaged 19.00 min (range: 17.50–21.50 min), and the total procedural time was 67.00 min (range: 62.00–69.50 min). In contrast, the five surgeons utilizing the 3D-printed simulation methodology achieved a reduced over-the-valve time of 5.22 min (range: 4.85–5.87 min), with a fluoroscopy duration of 15.00 min (range: 13.50–16.50 min), and a cumulative time of 57.00 min (range: 54.00–62.50 min) (Figure 3). Notably, there were no significant postoperative complications reported in the 3D-printed simulation group, including serious events such as coronary artery compression or valve embolization. However, in the non-3D-printed simulation cohort, two patients experienced hemoptysis postoperatively.

### 3.2. Experts Versus Young Surgeons in the Simulator 

**Figure 4:**
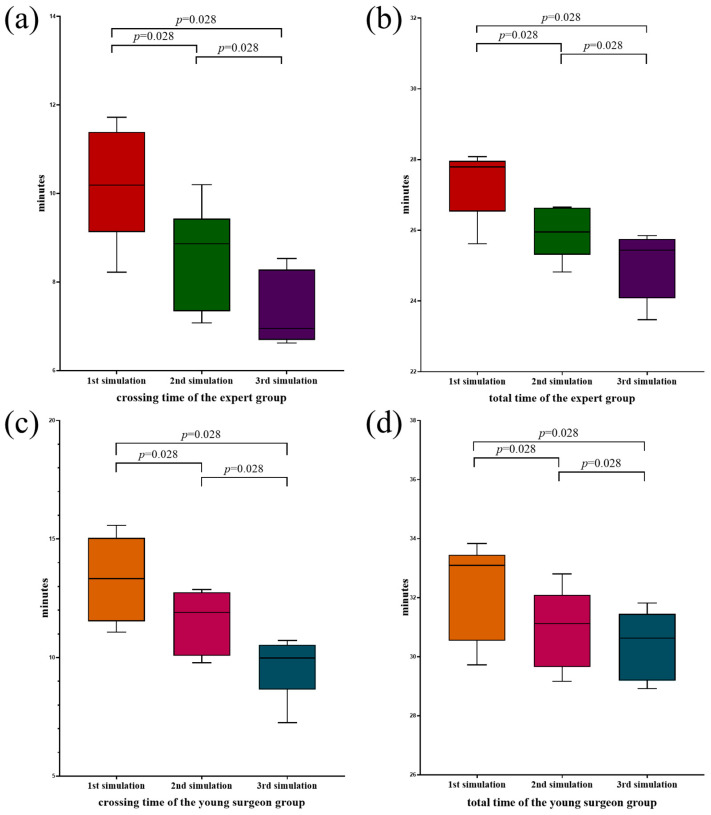
The outcomes of TPVR simulations. (**a**) A comparative analysis of the crossing-valve duration across three simulations within the expert cohort. (**b**) A comparative assessment of the total duration among the three simulations in the expert cohort. (**c**) A comparative evaluation of the crossing-valve duration across three simulations within the young surgeon cohort. (**d**) A comparative analysis of the overall duration among the three simulations in the young surgeon cohort. Statistical significance was determined using non-parametric tests and the corresponding *p*-values are reported.

Table 4 presents the baseline characteristics of the proceduralists involved in this study. Within the expert cohort, the average age was recorded at 42.00 years (range: 40.50–45.50 years), accompanied by an average professional experience of 12.50 years (range: 11.75–17.50 years), with 10.00 years (range: 8.50–11.25 years) specifically for interventional procedures. Conversely, in the young proceduralist cohort the mean age was noted to be 30.50 years (range: 29.75–31.50 years), with an average of 3.00 years (range: 2.00–4.00 years) of mean working experience and 1.50 years (range: 1.00–2.00 years) of experience in interventions, respectively. Six of the experts and six of the young procedural experts finished the three simulations successfully. The duration required for completing the simulations is detailed in Table 5. In the expert group, the crossing-valve time required for the first simulation was 10.19 min (range: 9.12–11.39 min), which decreased to 8.87 min (range: 7.34–9.44 min) and 6.95 min (range: 6.69–8.28 min) in the next two simulations. The total time for the three simulations showed a decreasing trend to 27.79 min (range: 26.53–27.96 min), 25.95 min (range: 25.31–26.64 min), and 25.44 min (range: 24.08–25.75 min), respectively. (Figure 4a,b). For the young proceduralist group, there was also a gradual trend of decreasing crossing-valve time and total time in the three simulations, with crossing-valve times of 13.33 min (range: 11.53–15.05 min), 11.91 min (range: 10.08–12.76 min), and 9.99 min (range: 8.65–10.54 min), and total times of 33.09 min (range: 30.55–33.45 min), 31.13 min (range: 29.66–32.10 min), and 30.64 min (range: 29.19–31.45 min), respectively (Figure 4c,d). From the above results, we observed a significant decrease in both crossing-valve and total time as the number of simulation training sessions increased. The reduction in time was independent of the experience level of the trainees.

## 4. Discussion

TPVR has been proven to be a safe, reliable, and effective treatment option for PR. For patients with severe PR after TOF treatment, TPVR can reduce the number of extracorporeal circulation procedures that these patients need throughout their lives, reduce the burden on patients, and improve their quality of life, and is expected to increase their life expectancy [15,16]. However, challenges remain due to the variability in the morphology and dimensions of the nRVOT after early TOF treatment. A study by Schievano et al. [17] performed 3D reconstruction and morphological classification of 83 patients with RVOT dysfunction, ultimately identifying five main RVOT morphological types. The study suggests that the understanding of RVOT morphology should be enhanced and combined with outflow tract diameter and compliance to optimize the selection process for patients undergoing TPVR. In this way, the proportion of patients suitable for transcatheter treatment can be increased and their clinical prognosis improved. At the same time, cardiovascular 3D printing has also made great progress. The use of multiple materials (silicone, resin, polyethylene, rubber of different hardness) to print the RV-PA complex can effectively restore the complex nRVOT structure of patients with PR and achieve highly accurate anatomical reduction. Studies have shown that personalized 3D-printed patient-specific RV-PA models can visualize anatomical structures, enabling more accurate surgical strategy formulation and valve model selection [18,19]. In addition, 3D-printed RV-PA models can be used to predict surgical risks, such as coronary artery compression and valve displacement [13,14].

Research has demonstrated that the advantages of simulation-based training have been substantiated through extensive meta-analyses, suggesting that such training can consistently influence outcomes associated with the knowledge and skills of trainees. Etami et al. [20] used 3D printing technology combined with flexible and transparent resin materials to create a coronary artery intubation simulator with high precision and realism. The simulator was evaluated by 12 experts and its applicability in all aspects was recognized, especially in education and safety. This result provides a new solution for cardiac intervention training, which can effectively reduce the risk during the learning process and improve the practical ability of students. Barabas et al. [21] used 3D printing technology to create hands-on surgical training tools in the education of congenital coarctation of the aorta, which can significantly improve medical students’ understanding of coarctation of the aorta and its surgical treatment options. This study highlights the potential of 3D printing technology in medical education, especially in the training of complex operations, to provide students with a more intuitive learning experience. Torres et al. [22] used 3D printing to develop a patient-specific simulator that is feasible in training endovascular repair of abdominal aortic aneurysms and significantly improves surgical performance and confidence in surgical residents. This study highlights the potential application of 3D printing technology in surgical training, which may provide a more economical and effective training method for future medical education. In short, the construction of an in vitro simulation platform based on 3D printing can greatly help improve the quality of training and technical skills of young doctors. However, the application of 3D printing technology in TPVR is currently mostly used as an intuitive and convenient surgical planning tool, especially in the selection of biological prostheses [23]. At present, there is a lack of sufficient evidence in the research on TPVR simulation training.

In this study, our innovation lies in being the first application of 3D printing technology-based simulation training in TPVR training. The findings of this study indicated that the group utilizing 3D printing simulation experienced a notable enhancement in several metrics, including operating duration, crossing-valve interval, DSA duration, radiation exposure, and incidence of complications, when contrasted with the group that did not employ 3D printing simulation. In addition, this study conducted a comparative study based on simulation training and found that with the increase in the number of simulations, the simulation proficiency of experts and young proceduralists improved, and the time required was significantly shortened. In conclusion, the findings of this research indicate that a three-dimensional (3D)-printed in vitro simulation platform serves as a valuable practical training resource for TPVR. The application of 3D printing technology in simulations has been widely used in the perioperative evaluation of TPVR, significantly contributing to the refinement and advancement of surgical methodologies. The patient-specific RV-PA model used in this study can also be used by surgeons in the preoperative treatment stage of clinical practice to better understand the patient’s anatomy. The application of this clinical implementation has the potential to decrease the occurrence of unexpected intraoperative complications, thereby leading to improved surgical outcomes. The swift advancement of imaging and 3D printing technologies has enabled the creation of 3D-printed models that can precisely depict a patient’s anatomical structure. This innovation provides surgeons with invaluable visual information in a controlled environment and has proven to be highly effective in addressing complex congenital heart diseases, valvular disorders, and various other medical conditions [24,25,26]. Customized models have the potential to enhance the interaction between surgeons and patients. Additionally, proceduralists and medical students could utilize in vitro simulations to replicate the surgical procedure [27]. It now takes only 6 h to reconstruct and print a patient’s specific RV-PA model, making it easy for the surgeon to assess each patient requiring TPVR preoperatively. In addition, for complex cases the simulator only needs to replace the RV-PA model of that patient to make preoperative simulation contacts to improve the success rate of surgery. This approach has the potential to not only educate surgeons and medical trainees but also to create individualized preoperative strategies for patients. Such personalized plans are crucial in enhancing both the safety and success rates of surgical procedures.

The limitations of this study are mainly reflected in the small sample size and the lack of clinical validation analysis. Although the results show the effectiveness of the TPVR simulator in training, the small sample size may lead to a decrease in the statistical power of the results, which affects the general applicability and promotion value. Therefore, future studies should consider increasing the sample size and conducting clinical validation to further confirm the role and impact of simulators in surgical training. Moreover, under the present circumstances the characteristics of polymer materials employed in 3D printing continue to pose challenges in achieving an optimal balance between elasticity and toughness. This limitation hampers the precision of forecasting certain complications during simulations. Nonetheless, there is no doubt that materials research will also be a major area of focus as safety requirements are constantly on the increase. In addition, although the TPVR simulator uses a circulatory pump to simulate the patient’s cardiac blood flow state, the current circulatory pump cannot meet the requirements of cardiac hemodynamics assessment, and the simulator needs to be further adjusted to meet higher clinical needs [28]. In recent years, significant advancements have been achieved in the field of 3D bioprinting [29]. As interdisciplinary collaboration advances within the realms of bioengineering, materials science, biology, and computer science, significant progress is anticipated in cardiovascular three-dimensional (3D) printing for simulation training and educational purposes. Despite the existing disparity between present capabilities and clinical applications, ongoing advancements are expected to yield novel breakthroughs in this domain.

## 5. Conclusions

This investigation introduces a novel training tool aimed at enhancing the instructional and training methodologies associated with TPVR. Patient-specific models can be used to effectively plan for the later stages of pre-surgery, and the performance of trained physicians after training not only improved, but was significantly better than untrained physicians, while helping to reduce radiation exposure and surgical risk. The utilization of three-dimensional (3D)-printed models in medical training significantly bolsters physicians’ comprehension of anatomical configurations and the pathophysiological characteristics associated with cardiovascular disorders. Additionally, it enhances their skills in procedural tasks, augments the overall training efficacy, reduces the learning curve, and enriches practical experience. While the current simulator may not provide a wholly authentic simulation experience, its dependable simulation quality indicates a strong potential for future clinical applications.

## Figures and Tables

**Table 1:** Patient characteristics.

Characteristics	Patient1	Patient2	Patient3	Patient4	Patient5	Patient6	Patient7	Patient8	Patient9	Patient10
Age, years/sex	19/M	30/F	37/F	52/F	23/M	48/F	12/M	22/F	31/F	42/M
Weight (kg)	68	62	60	55	78	53	51	68	58	81
Height (cm)	175	158	162	156	173	156	150	170	166	178
NYHA functional class	II	III	III	IV	III	IV	II	III	IV	IV
PR severity grade	4+	4+	4+	4+	4+	4+	4+	4+	4+	4+
Peak transpulmonary valve gradient	11	17	19	21	17	19	11	18	1 7	10
TR severity grade	1+	3+	3+	2+	3+	3+	2+	2+	3+	3+
RV–PA conduit length (mm)	58	52	57	55	59	55	48	53	56	62
nRVOT diameter (mm)	44	38	37	36	41	37	32	35	38	43
The narrowest plane/diameter (mm)	Distal MPA/23	Distal MPA/24	Mid MPA/28	Mid MPA/23	PA/ 33	Mid MPA/27	Distal MPA/24	Distal MPA/22	Mid MPA/26	PA/30

NYHA—New York Heart Association; PR—pulmonary regurgitation; TR—tricuspid regurgitation; RV–PA—right ventricle–pulmonary artery; nRVOT—native right ventricular outflow tract.

**Table 2:** Baseline information of experts in the 3D-printed simulation group and the non-3D-printed simulation group (*n* = 10).

Number	Sex	Age (Years)	Years for Proceduralist	Years for Intervention	Patient Number
A1	Male	46	16	11	1
A2	Male	42	13	7	2
A3	Male	43	15	10	3
A4	Male	39	12	8	4
A5	Male	38	11	9	5
B1	Male	42	13	8	6
B2	Male	40	10	9	7
B3	Male	45	16	12	8
B4	Male	37	10	8	9
B5	Male	43	11	7	10

Group A represents the 3D-printed simulation group; group B represents the non-3D-printed simulation group.

**Table 3:** Results of the 3D-printed simulation group versus the non-3D-printed simulation group.

Time	Crossing Valve	Fluoroscopy	Total	Residual PR	Complications
A1	5′13″	15′00″	53′00″	None	None
A2	4′55″	14′00″	57′00″	None	None
A3	5′40″	16′00″	62′00″	None	None
A4	4′47″	13′00″	55′00″	None	None
A5	6′05″	17′00″	63′00″	None	None
B1	5′55″	17′00″	61′00″	None	None
B2	7′13″	21′00″	71′00″	Trace	Hemoptysis
B3	6′43″	19′00″	68′00″	None	None
B4	6′20″	18′00″	67′00″	None	None
B5	8′11″	22′00″	63′00″	None	Hemoptysis

Group A represents the 3D-printed simulation group; group B represents the non-3D-printed simulation group.

**Table 4:** Baseline information of proceduralists in the expert group and the young proceduralist group (*n* = 12).

Number	Sex	Age (Years)	Years for Proceduralist	Years for Intervention
C1	Male	43	12	10
C2	Male	47	17	11
C3	Male	41	12	12
C4	Male	45	19	10
C5	Male	39	13	9
C6	Male	41	11	7
D1	Male	31	2	2
D2	Male	30	4	2
D3	Male	33	3	1
D4	Male	29	3	2
D5	Male	31	2	1
D6	Male	30	4	1

Group C represents the expert group; group D represents the young proceduralist group.

**Table 5:** Results of the expert group versus the young proceduralist group.

Rank	1st		2nd		3rd	
	Device	Total	Device	Total	Device	Total
C1	10′35″	27′49″	8′56″	26′07″	6′37″	25′43″
C2	9′48″	26′55″	9′11″	25′28″	7′06″	25′19″
C3	8′13″	25′37″	7′26″	24′49″	6′43″	24′17″
C4	11′17″	27′46″	8′48″	26′39″	8′12″	25′51″
C5	9′26″	26′50″	7′05″	25′47″	6′48″	23′28″
C6	11′43″	28′05″	10′12″	26′38″	8′32″	25′33″
D1	12′49″	33′19″	12′03″	31′26″	10′29″	30′51″
D2	11′41″	30′49″	9′47″	29′49″	7′15″	29′17″
D3	13′50″	32′53″	12′43″	30′49″	10′22″	30′25″
D4	11′05″	29′44″	10′11″	29′10″	9′36″	28′55″
D5	15′34″	33′50″	12′52″	32′48″	10′43″	31′49″
D6	14′52″	33′18″	11′46″	31′52″	9′07″	31′20″

Group C represents the expert group; group D represents the young proceduralist group.

## Data Availability

The data presented in this study are available on request from the corresponding author.

## References

[1] Basquin A., Pineau E., Galmiche L., Bonnet D., Sidi D., Boudjemline Y. (2010). Transcatheter Valve Insertion in a Model of Enlarged Right Ventricular Outflow Tracts. J. Thorac. Cardiovasc. Surg..

[2] Latus H., Stammermann J., Voges I., Waschulzik B., Gutberlet M., Diller G.P., Schranz D., Ewert P., Beerbaum P., Kühne T. (2022). Impact of Right Ventricular Pressure Load after Repair of Tetralogy of Fallot. J. Am. Heart Assoc..

[3] Álvarez-Fuente M., Toledano M., Garrido-Lestache E., Sánchez I., Molina I., Rivero N., García-Ormazábal I., Del Cerro M.J. (2023). Balloon-Expandable Pulmonary Valves for Patched or Native Right Ventricular Outflow Tracts. Pediatr. Cardiol..

[4] Jalal Z., Valdeolmillos E., Malekzadeh-Milani S., Eicken A., Georgiev S., Hofbeck M., Sieverding L., Gewillig M., Ovaert C., Bouvaist H. (2021). Mid-Term Outcomes Following Percutaneous Pulmonary Valve Implantation Using the “Folded Melody Valve” Technique. Circ. Cardiovasc. Interv..

[5] Martin M.H., Meadows J., McElhinney D.B., Goldstein B.H., Bergersen L., Qureshi A.M., Shahanavaz S., Aboulhosn J., Berman D., Peng L. (2018). Safety and Feasibility of Melody Transcatheter Pulmonary Valve Replacement in the Native Right Ventricular Outflow Tract: A Multicenter Pediatric Heart Network Scholar Study. JACC Cardiovasc. Interv..

[6] Guzeltas A., Tanidir I.C., Gokalp S., Topkarci M.A., Sahin M., Ergul Y. (2021). Implantation of the Edwards Sapien Xt and Sapien 3 Valves for Pulmonary Position in Enlarged Native Right Ventricular Outflow Tract. Anatol. J. Cardiol..

[7] Tannous P., Nugent A. (2021). Transcatheter Pulmonary Valve Replacement in Native and Nonconduit Right Ventricle Outflow Tracts. J. Thorac. Cardiovasc. Surg..

[8] Park W.Y., Kim G.B., Lee S.Y., Kim A.Y., Choi J.Y., Jang S.I., Kim S.H., Cha S.G., Wang J.K., Lin M.T. (2024). The Adaptability of the Pulsta Valve to the Diverse Main Pulmonary Artery Shape of Native Right Ventricular Outflow Tract Disease. Catheter. Cardiovasc. Interv..

[9] Corrigan F.E., Gleason P.T., Condado J.F., Lisko J.C., Chen J.H., Kamioka N., Keegan P., Howell S., Clements S.D., Babaliaros V.C. (2019). Imaging for Predicting, Detecting, and managing complications After transcatheter aortic Valve Replacement. JACC Cardiovasc. Imaging.

[10] Fan Y., Wong R.H.L., Lee A.P. (2019). Three-Dimensional Printing in Structural Heart Disease and Intervention. Ann. Transl. Med..

[11] Pluchinotta F.R., Sturla F., Caimi A., Giugno L., Chessa M., Giamberti A., Votta E., Redaelli A., Carminati M. (2020). 3-Dimensional Personalized Planning for Transcatheter Pulmonary Valve Implantation in a Dysfunctional Right Ventricular Outflow Tract. Int. J. Cardiol..

[12] Schievano S., Migliavacca F., Coats L., Khambadkone S., Carminati M., Wilson N., Deanfield J.E., Bonhoeffer P., Taylor A.M. (2007). Percutaneous Pulmonary Valve Implantation Based on Rapid Prototyping of Right Ventricular Outflow Tract and Pulmonary Trunk from Mr Data. Radiology.

[13] Han Y., Shao Z., Sun Z., Han Y., Xu H., Song S., Pan X., de Jaegere P.P.T., Fan T., Zhang G. (2024). In Vitro Bench Testing Using Patient-Specific 3d Models for Percutaneous Pulmonary Valve Implantation with Venus P-Valve. Chin. Med. J..

[14] Wang Y., Jin P., Meng X., Li L., Mao Y., Zheng M., Liu L., Liu Y., Yang J. (2023). Treatment of Severe Pulmonary Regurgitation in Enlarged Native Right Ventricular Outflow Tracts: Transcatheter Pulmonary Valve Replacement with Three-Dimensional Printing Guidance. Bioengineering.

[15] Müller J., Engelhardt A., Fratz S., Eicken A., Ewert P., Hager A. (2014). Improved Exercise Performance and Quality of Life after Percutaneous Pulmonary Valve Implantation. Int. J. Cardiol..

[16] Cheatham J.P., Hellenbrand W.E., Zahn E.M., Jones T.K., Berman D.P., Vincent J.A., McElhinney D.B. (2015). Clinical and Hemodynamic Outcomes up to 7 Years after Transcatheter Pulmonary Valve Replacement in the Us Melody Valve Investigational Device Exemption Trial. Circulation.

[17] Schievano S., Coats L., Migliavacca F., Norman W., Frigiola A., Deanfield J., Bonhoeffer P., Taylor A.M. (2007). Variations in Right Ventricular Outflow Tract Morphology Following Repair of Congenital Heart Disease: Implications for Percutaneous Pulmonary Valve Implantation. J. Cardiovasc. Magn. Reson..

[18] Zhou D., Pan W., Jilaihawi H., Zhang G., Feng Y., Pan X., Liu J., Yu S., Cao Q., Ge J. (2019). A Self-Expanding Percutaneous Valve for Patients with Pulmonary Regurgitation and an Enlarged Native Right Ventricular Outflow Tract: One-Year Results. EuroIntervention.

[19] Odemis E., Aka I.B., Ali M.H.A., Gumus T., Pekkan K. (2023). Optimizing Percutaneous Pulmonary Valve Implantation with Patient-Specific 3d-Printed Pulmonary Artery Models and Hemodynamic Assessment. Front. Cardiovasc. Med..

[20] Etami H.V., Rismawanti R.I., Hanifah V.A.N., Herianto H., Yanuar Y., Kuswanto D., Anggrahini D.W., Gharini P.P.R. (2022). Ct-Derived 3d Printing for Coronary Artery Cannulation Simulator Design Manufacturing. Bioengineering.

[21] Barabas I.J., Vegh D., Bottlik O., Kreuter P., Hartyanszky I., Merkely B., Palkovics D. (2024). The Role of 3d Technology in the Practical Education of Congenital Coarctation and Its Treatment-a Feasibility Pilot Study. BMC Med. Educ..

[22] Torres I.O., De Luccia N. (2017). A Simulator for Training in Endovascular Aneurysm Repair: The Use of Three Dimensional Printers. Eur. J. Vasc. Endovasc. Surg..

[23] Chessa M., Giugno L., Butera G., Carminati M. (2016). Multi-Modal Imaging Support in a Staging Percutaneous Pulmonary Valve Implantation. Eur. Heart J..

[24] Qian Z., Wang K., Liu S., Zhou X., Rajagopal V., Meduri C., Kauten J.R., Chang Y.H., Wu C., Zhang C. (2017). Quantitative Prediction of Paravalvular leak in Transcatheter Aortic valve Replacement Based On tissue-Mimicking 3d Printing. JACC Cardiovasc. Imaging.

[25] Santoro G., Pizzuto A., Rizza A., Cuman M., Federici D., Cantinotti M., Pak V., Clemente A., Celi S. (2021). Transcatheter Treatment of “Complex” Aortic Coarctation Guided by Printed 3d Model. JACC Case Rep..

[26] Mao Y., Liu Y., Zhai M., Yang J. (2023). Application of and Prospects for 3-Dimensional Printing in Transcatheter Mitral Valve Interventions. Rev. Cardiovasc. Med..

[27] Ding P., Li L., Liu Y., Jin P., Tang J., Yang J. (2021). Three-Dimensional Printing for Heart Diseases: Clinical Application Review. Biodes Manuf..

[28] Marques D.M.C., Silva J.C., Serro A.P., Cabral J.M.S., Sanjuan-Alberte P., Ferreira F.C. (2022). 3d Bioprinting of Novel Κ-Carrageenan Bioinks: An Algae-Derived Polysaccharide. Bioengineering.

[29] Mayfield C.K., Ayad M., Lechtholz-Zey E., Chen Y., Lieberman J.R. (2022). 3d-Printing for Critical Sized Bone Defects: Current Concepts and Future Directions. Bioengineering.

